# Phenolic Profiles, Antioxidant Capacities, and Inhibitory Effects on Digestive Enzymes of Different Kiwifruits

**DOI:** 10.3390/molecules23112957

**Published:** 2018-11-13

**Authors:** Hong-Yi Li, Qin Yuan, Yu-Ling Yang, Qiao-Hong Han, Jing-Liu He, Li Zhao, Qing Zhang, Shu-Xiang Liu, De-Rong Lin, Ding-Tao Wu, Wen Qin

**Affiliations:** College of Food Science, Sichuan Agricultural University, Ya’an 625014, Sichuan, China; lhongyi45@163.com (H.-Y.L.); 18983426354@163.com (Q.Y.); yangyulingmie@163.com (Y.-L.Y.); hqhlzy@163.com (Q.-H.H.); hjingliu@163.com (J.-L.H.); zhaoli0608@126.com (L.Z.); zhangqing@sicau.edu.cn (Q.Z.); sliu@sicau.edu.cn (S.-X.L.); lindr2018@sicau.edu.cn (D.-R.L.)

**Keywords:** kiwifruit, phenolic compounds, HPLC analysis, antioxidant capacity, enzyme inhibition

## Abstract

In order to obtain high-quality kiwifruits with health-promoting characteristics, physicochemical properties, phenolic profiles, antioxidant capacities, and inhibitory effects on digestive enzymes (pancreatic lipase and α-glucosidase), of fourteen different types of kiwifruit obtained from China were systematically investigated and compared. Noticeable variations in the fruits’ physicochemical properties and phenolic profiles were observed among them. The total phenolic content of *Actinidia chinensis* cv. Hongshi, *A. chinensis* cv. Jinshi, and *A. chinensis* cv. Jinlong were 16.52 ± 0.26 mg GAE/g DW (dry weight), 13.38 ± 0.20 mg GAE/g DW, and 11.02 ± 0.05 mg GAE/g DW, respectively, which were much higher than those of the other tested kiwifruits. According to high performance liquid chromatography (HPLC) analysis, phenolic compounds, including procyanidin B1, procyanidin B2, (−)-epicatechin, chlorogenic acid, gallic acid, and quercetin-3-rhamnoside, were found to be the major compounds in kiwifruits, while procyanidin B1, procyanidin B2, and chlorogenic acid were the most abundant phenolic compounds. Furthermore, all the tested kiwifruits exerted remarkable antioxidant capacities and inhibitory effects on pancreatic lipase and α-glucosidase. Indeed, *A. chinensis* cv. Hongshi, *Actinidia chinensis* cv. Jinshi, and *Actinidia chinensis* cv. Jinlong exhibited much better antioxidant capacities and inhibitory effects on digestive enzymes than those of the other tested kiwifruits. Particularly, *A. polygama* showed the highest inhibitory activity on α-glucosidase. Therefore, *Actinidia chinensis* cv. Hongshi, *Actinidia chinensis* cv. Jinshi, and *Actinidia chinensis* cv. Jinlong, as well as *A. polygama* could be important dietary sources of natural antioxidants and natural inhibitors against pancreatic lipase and α-glucosidase, which is helpful for meeting the growing demand for high-quality kiwifruits with health-promoting characteristics in China.

## 1. Introduction

Epidemiological studies have shown that a diet rich in fruits is associated with a lower risk of many diseases, such as cardiovascular diseases, diabetes, and even cancer [1,2,3]. The health benefits of fruits are partially attributed to the high content of bioactive components, such as phenolics and ascorbic acid [4,5]. Growing evidences indicate that the dietary consumption of phenolic-rich fruits can protect against oxidative damage [6,7]. Moreover, it is increasingly documented that phenolic compounds can also affect the digestive enzymes involved in the hydrolysis of dietary carbohydrates and fats [8]. Pancreatic lipase and α-glucosidase are key enzymes responsible for the hydrolysis of dietary fats and carbohydrates, respectively [8,9]. The inhibition of pancreatic lipase and α-glucosidase by phenolic-rich fruits may offer a natural dietary approach for preventing postprandial hyperglycemia and obesity [8,10]. Therefore, the dietary consumption of fruits seems to be a reasonable option for the prevention and treatment of oxidative damage, obesity, and type II diabetes.

**Figure 1:**
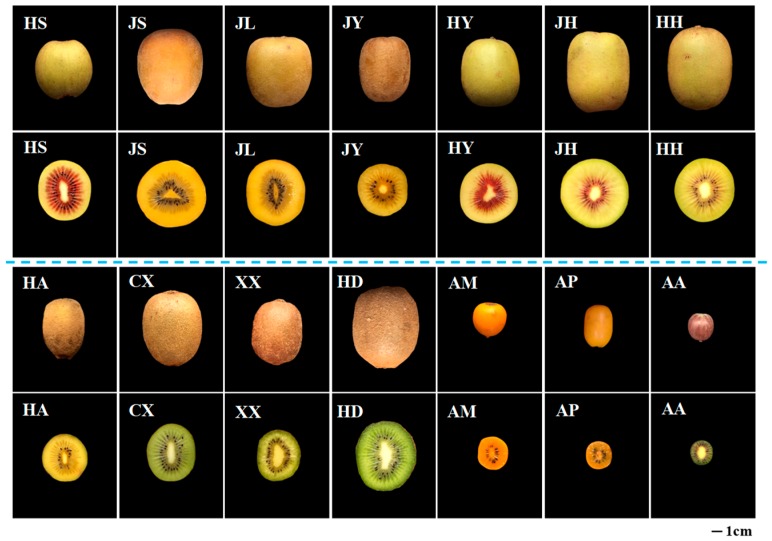
Morphological characteristics of representative kiwifruits collected in China. **HS**, *Actinidia chinensis* cv. Hongshi; **JS**, *A. chinensis* cv. Jinshi; **JL**, *A. chinensis* cv. Jinlong; **HY**, *A. chinensis* cv. Hongyang; **JH**, *A. chinensis* cv. Jinhong; **HH**, *A. chinensis* cv. Honghua; **HA**, *A. chinensis* cv. Hort16A; **CX**, *A. deliciosa* cv. Cuixiang; **XX**, *A. deliciosa* cv. Xuxiang; **HD**, *A. deliciosa* cv. Hayward; **AM**, *A. macrosperma*; **AP**, *A. polygama*; **AA**, *A. arguta*.

Kiwifruit is one of the most popular fruits in the world because of its flavor and health benefits [11,12]. The world production of kiwifruit was around 3,447,604 tons in 2016, accounting for around 0.5% of the world’s total fruit production. Indeed, China is the main producer of kiwifruit, accounting for 1.84 million tons and 53.4% of worldwide kiwifruit production [13]. In the last three decades, kiwifruit production has grown greatly to around 3.5 times in size. This tendency shows that kiwifruit is becoming more popular, probably due to their health benefits [13]. It is widely believed that the consumption of kiwifruits has a preventive effect against cardiovascular disease and obesity [1,8]. Indeed, it is confirmed that kiwifruits have various biological activities [13], such as antioxidant [14], anti-diabetes [8,14], anti-obesity [8,13], anti-proliferative [2], and anti-inflammatory activities [15]. Generally, phenolic compounds and ascorbic acid are considered the main bioactive components in kiwifruit, which are responsible for its various bioactivities [13,14]. When compared with orange, apple, peach, pear, banana, pineapple, red grapefruit, and other fruits, kiwifruits exhibit higher content of total phenolic compounds and higher antioxidant capacity [8]. Additionally, one kiwifruit can fulfill 85% of our daily ascorbic acid requirements [13]. The dietary consumption of kiwifruits makes a substantial contribution to its health-promoting effects. Furthermore, the phenolic compounds and bioactivities of kiwifruits are influenced by species and cultivars [14]. Regarding the genus of kiwifruit, there are more than 70 species in the world [16], and different kiwifruit cultivars have been cultivated during the past several decades, especially in New Zealand, Poland, and Greece. Indeed, different kiwifruit species and many new kiwifruit cultivars have also been widely cultivated and developed in China (Figure 1), respectively [17]. However, there are limited reports on the comparison of phenolic profiles and bioactive properties of different kiwifruit species and newly developed cultivars collected in China.

Nowadays, with improvements in quality of life, consumers are attaching increasing importance to health. An increasing number of consumers are not only searching for a kiwifruit cultivar which is sweet and has good flavor, but also health-promoting compounds, such as phenolic compounds [17]. Therefore, it is necessary to evaluate and compare the bioactive components and properties of different kiwifruits, which is helpful for meeting the growing demand for high-quality fruits with health-promoting characteristics in China. However, the biological activities of different kiwifruit species and newly developed cultivars collected in China have seldom been evaluated. Moreover, identification and quantification of phenolic profiles of different kiwifruits are critical starting points for assessing their biological properties [14]. Therefore, in this study, in order to obtain high-quality kiwifruits with health-promoting characteristics, the physicochemical properties, phenolic profiles, antioxidant capacities, and inhibitory effects on digestive enzymes (pancreatic lipase and α-glucosidase) of different kiwifruit species and newly developed cultivars collected in China were systematically investigated and compared.

## 2. Results and Discussion

### 2.1. Physicochemical Properties of Different Kiwifruits

The physicochemical properties of different kiwifruits, including the fresh weight per fruit, soluble solids content (SSC), and dry matter content, are shown in Table 1. Results showed that significant (*p* < 0.05) differences in the fresh weight per fruit, dry matter, and SSC were found among the tested kiwifruits. Briefly, the fresh weight per fruit ranged from 7.10 ± 0.37 g (*A. arguta*) to 133.74 ± 5.66 g (*A. chinensis* cv. Jinlong). The fresh weights per fruit of *A. macrosperma*, *A. polygama*, and *A. arguta* were much lower than those of *A. chinensis* and *A. deliciosa*, which is a similar result to previous studies [18]. Generally, the SSC consists of sugar, acid, vitamins, some minerals, and other soluble solid content, which is an important index of fruit taste quality and consumer acceptability [14,19]. The SSC ranged from 13.1% (*A. chinensis* cv. Jinyan) to 17.6% (*A. chinensis* cv. Hongyang), which is in accordance with a previous study [14]. *A. chinensis* cv. Hongyang, *A. chinensis* cv. Hongshi, *A. chinensis* cv. Jinshi, *A. chinensis* cv. Jinlong, and *A. chinensis* cv. Jinhong contained much more SSC (>16.1) than those of the other tested kiwifruits. According to some previous studies, the SSC of kiwifruits could be influenced by different environmental conditions and species [20,21]. Moreover, the dry matter of 14 different kiwifruits varied from 13.48% (*A. deliciosa* cv. Hayward) to 20.35% (*A. arguta*).

Furthermore, the content of ascorbic acid is a preferential factor for the evaluation of the quality of fruits [22]. Kiwifruits may be considered a good source of ascorbic acid. As shown in Table 1, the ascorbic acid content in different kiwifruits varied from 51.32 ± 0.42 to 390.68 ± 4.24 mg/100 g FW (fresh weight). Briefly, the ascorbic acid content of newly developed *A. chinensis* cv. Hongshi, *A. chinensis* cv. Jinshi, and *A. chinensis* cv. Jinlong were 390.68 ± 4.24 mg/100 g FW, 235.30 ± 8.27 mg/100 g FW, and 335.06 ± 10.39 mg/100 g FW, respectively, which were much higher than that of the most commercialized *A. deliciosa* cv. Hayward (59.32 ± 1.89 mg/100 g FW) [7,17]. The content of ascorbic acid was not only strongly affected by type of species, but also by different cultivars [1,14].

### 2.2. Phenolic Profiles of Different Kiwifruits

Phenolic compounds are considered the main bioactive components in kiwifruits [14]. However, the phenolic compounds of different kiwifruit species and newly developed cultivars collected in China have seldom been evaluated. As shown in Table 2, the total phenolic content (TPC) among the fourteen different kiwifruits differed significantly (*p* < 0.05), ranging from 3.75 ± 0.09 mg GAE/g DW to 16.52 ± 0.26 mg GAE/g DW. Results showed that the TPC of different kiwifruits was significantly affected by different species and cultivars. Briefly, the total phenolic content of newly developed *A. chinensis* cv. Hongshi, *A. chinensis* cv. Jinshi, and *A. chinensis* cv. Jinlong were 16.52 ± 0.26 mg GAE/g DW, 13.38 ± 0.20 mg GAE/g DW, and 11.02 ± 0.05 mg GAE/g DW, respectively, which were significantly (*p* < 0.05) higher than those of the other tested kiwifruits. However, the lowest level of TPC was found in the most commercialized *A. deliciosa* cv. Hayward (3.75 ± 0.09 mg GAE/g DW). Similar studies also showed that the total phenolic content in kiwifruits with red flesh (*A. chinensis* cv. Hongyang) and yellow flesh (*A. deliciosa* cv. Jinkui) were higher than that of kiwifruits with green flesh (*A. deliciosa* cv. Hayward) [1]. Generally, the genus and cultivar of kiwifruit, climatic conditions, harvest time, and extraction processes could affect the content of phenolic compounds [14,23,24].

**Figure 2:**
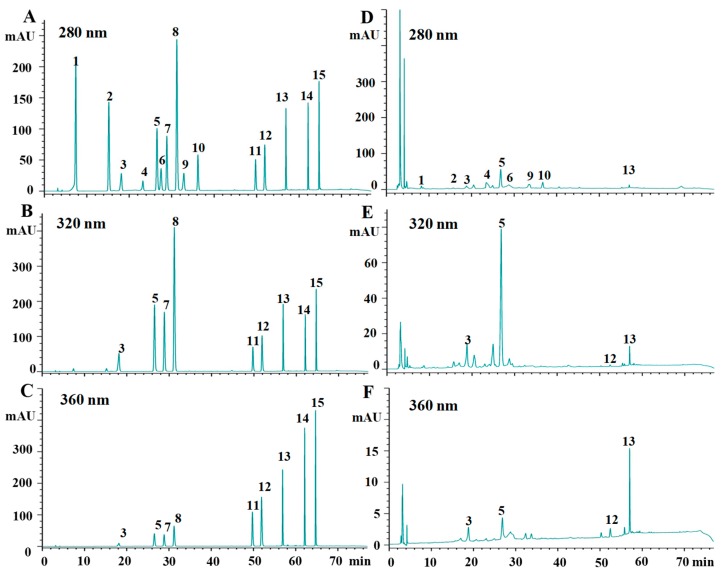
HPLC chromatograms of mixed standards (**A**–**C**) and representative phenolic profiles of *A. chinensis* cv. Hongshi cultivar (**D**–**F**). **1**, gallic acid; **2**, protocatechuic acid; **3**, neochlorogenic acid; **4**, procyanidin B1; **5**, chlorogenic acid; **6**, (+)-catechin; **7**, cryptochlorogenic acid; **8**, caffeic acid; **9**, procyanidin B2; **10**, (−)-epicatechin; **11**, rutin; **12**, quercetin-3-*O*-glucoside; **13**, quercetin-3-rhamnoside; **14**, quercetin; **15**, kaempferol. Detection was made at 280 nm for flavan-3-ols and hydroxybenzoic acids, 320 nm for hydroxycinnamic acids, and 360 nm for flavonols.

Furthermore, in order to properly understand the differences between individual phenolic compounds in tested kiwifruits, the HPLC-DAD analysis was performed. Previous studies have shown that flavan-3-ols (such as (−)-epicatechin, (+)-catechin), procyanidin B1, and procyanidin B2), hydroxybenzoic acids (such as gallic acid and protocatechuic acid), hydroxycinnamic acids (such as chlorogenic acid, neochlorogenic acid, and caffeic acid), and flavonols (such as quercetin-3-rhamnoside, quercetin-3-*O*-glucoside, and rutin) have been identified and found as the major phenolic compounds in kiwifruits [14,17,21,25]. Therefore, a total of fifteen commercially available phenolic compounds, including (+)-catechin, (−)-epicatechin, procyanidin B1, procyanidin B2, quercetin, rutin, kaempferol, quercetin-3-*O*-glucoside, quercetin-3-rhamnoside, chlorogenic acid, neochlorogenic acid, cryptochlorogenic acid, caffeic acid, gallic acid, and protocatechuic acid were selected and investigated in each kiwifruit extract according to previous studies. Figure 2 shows the chromatograms of the mixed standards and representative phenolic profiles of *A. chinensis* cv. Hongshi. The calibration data (regression equation, linear range, and R^2^) and limits of detection for the fifteen phenolic compounds investigated in kiwifruits are summarized in Table 3. Results showed that the developed HPLC method was suitable for the qualitative and quantitative analysis of phenolic compounds in different kiwifruits. A total of twelve phenolic compounds, including procyanidin B1, procyanidin B2, (−)-epicatechin, (+)-catechin, gallic acid, protocatechuic acid, neochlorogenic acid, chlorogenic acid, caffeic acid, rutin, quercetin-3-*O*-glucoside, and quercetin-3-rhamnoside, were identified in different species and cultivars of kiwifruits based on their HPLC retention time and UV spectra, which is similar with previous studies [14,17,21,25]. The content of individual phenolic compounds in different kiwifruits are summarized in Table 4. However, as shown in Table 4, the type and content of phenolic compounds varied significantly by species and cultivars.

As shown in Table 4, four flavan-3-ols, including two monomeric flavan-3-ols ((−)-epicatechin and (+)-catechin) and two dimeric procyanidins (procyanidin B1 and procyanidin B2), were detected in different species and cultivars of kiwifruits. Results are in accordance with previous studies [17,25]. The total flavan-3-ols content in different kiwifruits ranged from 96.07 μg/g DW (*A. deliciosa* cv. Cuixiang) to 823.61 μg/g DW (*A. chinensis* cv. Hongshi). The highest content of total flavan-3-ols determined in *A. chinensis* cv. Hongshi may be attributed to its large part of red flesh, as shown in Figure 1. Briefly, the content of (−)-epicatechin varied significantly among the tested kiwifruits. The highest content of (−)-epicatechin was measured in *A. chinensis* cv. Hongshi (162.61 ± 0.99 μg/g DW). However, (−)-epicatechin was not found in *A. chinensis* cv. Honghua, *A. deliciosa* cv. Hayward, and *A. arguta*. As for (+)-catechin, it was only found in *A. chinensis* cv. Hongshi, *A. deliciosa* cv. Xuxiang, *A. macrosperma*, *A. polygama*, and *A. arguta*. The highest content of (+)-catechin was measured in *A. arguta* (132.08 ± 1.16 μg/g DW). The procyanidin B1 predominated among the flavan-3-ols. Procyanidin B1 content varied significantly (*p* < 0.05) among different species and cultivars. The highest content of procyanidin B1 was measured in *A. chinensis* cv. Hongshi (446.81 ± 1.51 μg/g DW), which was two times higher than that of the most commercialized *A. deliciosa* cv. Hayward (203.68 ± 4.27 μg/g DW). Similarly to procyanidin B1, procyanidin B2 content also varied greatly among the tested kiwifruits, and the highest content was also measured in *A. chinensis* cv. Hongshi (182.11 ± 1.16 μg/g DW). However, procyanidin B2 was not found in *A. chinensis* cv. Jinshi, Jinhong, and Honghua. Finally, results indicated that (−)-epicatechin and procyanidin B1 were the main flavan-3-ols in kiwifruits, which is in accordance with previous reports [17,25]. Results showed that the types and content of flavan-3-ols in kiwifruits were significantly affected by species and cultivars.

Phenolic acids, including two hydroxybenzoic acids (gallic acid and protocatechuic acid) and three hydroxycinnamic acids (chlorogenic acid, neochlorogenic acid, and caffeic acid), were found in different species and cultivars of kiwifruits. The content of total phenolic acids ranged from 26.58 μg/g DW (*A. deliciosa* cv. Cuixiang) to 315.76 μg/g DW (*A. chinensis* cv. Hongshi). Briefly, as shown in Table 4, chlorogenic acid, as a caffeoylquinic acid, was not only the main individual hydroxycinnamic acid in different kiwifruits, but also the main individual phenolic compound in different kiwifruits, which is in accordance with previous studies [17,26]. The content of chlorogenic acid differed significantly (*p* < 0.05) among the tested kiwifruits, ranging from 7.70 ± 0.04 μg/g DW (*A. arguta*) to 235.75 ± 5.44 μg/g DW (*A. chinensis* cv. Hongshi). Meanwhile, the content of neochlorogenic acid also varied significantly (*p* < 0.05) among the tested kiwifruits. The highest content of neochlorogenic acid was detected in *A. chinensis* cv. Jinlong (133.72 ± 3.98 μg/g DW). However, neochlorogenic acid was not found in *A. deliciosa* cv. Cuixiang, *A. deliciosa* cv. Xuxiang, *A. deliciosa* cv. Hayward, *A. macrosperma*, and *A. polygama*. Furthermore, caffeic acid was only found in *A. macrosperma*, *A. polygama*, and *A. arguta*, which indicated that the species significantly affected the types of phenolic compounds in kiwifruits. Moreover, gallic acid was the main individual hydroxybenzoic acid found in different kiwifruits, which is in accordance with a previous study [17]. The content of gallic acid varied significantly (*p* < 0.05) among the tested kiwifruits. The top three samples were *A. chinensis* cv. Jinshi (53.76 ± 0.43 μg/g DW) > *A. chinensis* cv. Jinyan (49.13 ± 0.27 μg/g DW) > *A. chinensis* cv. Jinlong (26.69 ± 0.45 μg/g DW). However, gallic acid was not detected in *A. macrosperma* and *A. arguta*. Additionally, protocatechuic acid was only detected in *A. chinensis* cv. Hongshi, *A. chinensis* cv. Jinshi, and *A. polygama*, and the highest content was measured in *A. chinensis* cv. Hongshi (15.76 ± 0.18 μg/g DW).

Furthermore, three flavonols, including quercetin-3-rhamnoside, quercetin-3-*O*-glucoside, and rutin were identified in the tested kiwifruits. The content of total flavonols varied greatly among the tested kiwifruits. The main flavonol of the tested kiwifruits was quercetin-3-rhamnoside, which is consistent with previous studies [25,27]. The highest content of quercetin-3-rhamnoside was detected in *A. chinensis* cv. Hongshi (41.94 ± 1.44 μg/g DW). However, quercetin-3-rhamnoside was not detected in *A. chinensis* cv. Jinlong and *A. deliciosa* cv. Hayward. Moreover, rutin was only found in *A. macrosperma*, *A. polygama*, and *A. arguta*. Similarly to rutin, quercetin-3-*O*-glucoside was also only detected in *A. chinensis* cv. Hongshi, *A. deliciosa* cv. Xuxiang, *A. polygama*, and *A. arguta*.

### 2.3. Antioxidant Capacities of Different Kiwifruits

The contribution of kiwifruits to health improvement has been partly attributed to their antioxidant capacity [13]. Generally, the antioxidant capacity of fruits is affected by different mechanisms of action of their antioxidant constituents [8]. Therefore, the antioxidant capacity was evaluated by different methods. Table 2 also summarized the antioxidant capacities of different kiwifruits. Significant differences (*p* < 0.05) were found in the tested kiwifruits. Briefly, the antioxidant capacities ranged from 32.95 ± 0.29 μmol Trolox/g DW (*A. deliciosa* cv. Hayward) to 160.36 ± 6.15 μmol Trolox/g DW (*A. chinensis* cv. Hongshi) in ABTS assay, from 13.12 ± 0.07 μmol Trolox/g DW (*A. chinensis* cv. Jinhong) to 87.38 ± 4.32 μmol Trolox/g DW (*A. chinensis* cv. Hongshi) in DPPH assay, and from 27.24 ± 0.41 μmol Trolox/g DW (*A. chinensis* cv. Jinhong) to 149.97 ± 6.98 μmol Trolox/g DW (*A. chinensis* cv. Hongshi) in FRAP assay, respectively. These results are similar with previous studies [24,28]. *A. chinensis* cv. Hongshi showed the highest antioxidant capacities, followed by *A. chinensis* cv. Jinshi and *A. chinensis* cv. Jinlong, while *A. deliciosa* cv. Hayward showed the lowest antioxidant capacities, regardless of assay methods. The differences in antioxidant capacity of the tested kiwifruits could be preliminarily attributed to the different content of phenolic compounds and ascorbic acid. As shown in Table 2, the antioxidant capacities of the tested kiwifruits evaluated by ABTS assay, DPPH assay, and FRAP assay were positively correlated with the TPC, respectively. Generally, phenolic compounds and ascorbic acid play important roles in fruits’ antioxidant capacities [14]. Previous studies have shown that both total polyphenols and ascorbic acid are major contributors to the total antioxidant capacity in kiwifruits [28]. In particular, it was reported that chlorogenic acid showed a better correlation with antioxidant activity (DPPH, ABTS, and FRAP) [29]. Thus, it seems likely that the high content of chlorogenic acid could be one of the major contributors to the antioxidant capacities of kiwifruits. Results suggested that *A. chinensis* cv. Hongshi, *A. chinensis* cv. Jinshi, and *A. chinensis* cv. Jinlong could be potential resources of antioxidants for the production of health-benefiting products.

### 2.4. Inhibitory Effects on Digestive Enzymes of Different Kiwifruits

**Figure 3:**
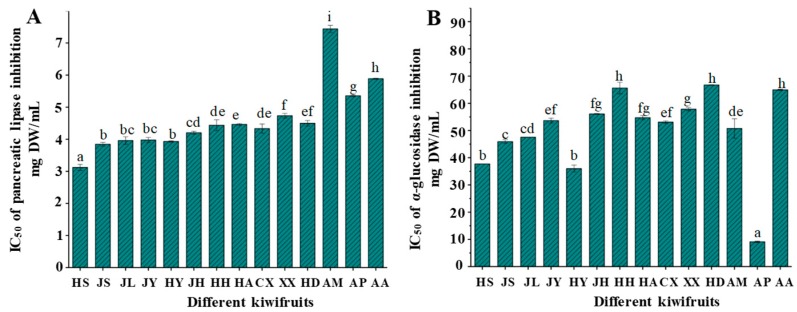
Inhibitory activities on pancreatic lipase (**A**) and α-glucosidase (**B**) of different kiwifruits. All experiments were conducted in triplicate. Values represent mean ± standard deviation, and the error bars are standard deviations. Different letters (a–i) in the same column indicate significant differences at *p* < 0.05 determined by ANOVA, followed by Duncan’s test. The sample codes are the same as in Table 1.

Pancreatic lipase is the most important enzymes responsible for triglyceride digestion. Consequently, the suppression and delay of triglyceride digestion and absorption through inhibition of lipase is a key approach to the control of hyperlipidaemia and obesity [10]. Previous studies have shown that the consumption of kiwifruit plays a significant role in the levels of plasmatic lipids [13], and that kiwifruit extracts exhibit a strong inhibitory effect on pancreatic lipase [8]. However, the inhibitory effects on lipase of different species and cultivars of kiwifruits collected in China have seldom been investigated and compared. As shown in Figure 3A, significant differences (*p* < 0.05) were found among the tested kiwifruits in the inhibitory activities toward pancreatic lipase. The IC_50_ values of inhibitory effects on pancreatic lipase varied from 3.12 ± 0.09 mg/mL to 7.44 ± 0.11 mg/mL among the tested kiwifruits. The difference in IC_50_ values may have resulted from the different levels of TPC in kiwifruits, which suggests that the inhibitory effects on pancreatic lipase were associated with the high content of phenolic compounds [9]. Among the tested kiwifruits, *A. chinensis* cv. Hongshi showed the highest pancreatic lipase inhibitory activity, whereas the lowest activity was measured in *A. macrosperma*. In addition, *A. chinensis* and *A. deliciosa* showed more potent inhibitory effects on pancreatic lipase than other species (*A. macrosperma, A. polygama*, and *A. arguta*). Furthermore, the inhibitory effects on pancreatic lipase of the tested kiwifruits (except for *A. macrosperma*) still presented much lower IC_50_ values when compared with the commercial orlistat drug (IC_50_ = 6.34 mg/mL). Previous studies have reported that proanthocyanidins are effective lipase inhibitors in grape seed extract and strawberry extract [30,31]. In addition, previous studies have shown that quercetin and its derivatives exert an inhibition effect on pancreatic lipase [32,33]. Therefore, it seems likely that proanthocyanidins (procyanidin B1 and procyanidin B2) and quercetin derivatives (quercetin-3-rhamnoside and quercetin-3-*O*-glucoside) present in kiwifruits could be the major contributors toward the pancreatic lipase inhibition effects of kiwifruits due to their high content.

α-Glucosidase is a key enzyme responsible for the breakdown of oligosaccharides and disaccharides into monosaccharides suitable for absorption. Therefore, the inhibition of α-glucosidase is one of the main strategies to counteract metabolic alterations related to hyperglycaemia and type II diabetes [34]. Previous studies have shown that the kiwifruit extract exhibits a strong inhibitory effect on α-glucosidase [8,14]. However, different kiwifruits’ inhibitory effects on α-glucosidase have seldom been investigated. As shown in Figure 3B, significant differences (*p* < 0.05) were found among the tested kiwifruits in the inhibitory activities toward α-glucosidase. There was a wide range of α-glucosidase inhibition, with the IC_50_ values ranging from 9.11 ± 0.24 mg/mL to 66.73 ± 0.09 mg/mL. Particularly, *A. polygama* showed the highest inhibitory activity on α-glucosidase, which might be attributed to the high content of quercetin-3-*O*-glucoside. A previous study showed that the flavonols, especially quercetin-3-*O*-glucoside, were found to possess high inhibition activity on α-glucosidase [9]. Moreover, previous studies have also indicated that chlorogenic acid and its structural isomer are major contributors to the inhibitory activities on α-glucosidase [32,35]. The high content of chlorogenic acid and neochlorogenic acid in kiwifruits may explain the α-glucosidase inhibitory effect of kiwifruits. Caffeic acid and catechin might also play important roles in α-glucosidase inhibition effects [29,32,35]. The order of the most potent α-glucosidase inhibitors was as follows: *A. polygama* > *A. chinensis* cv. Hongshi > *A. chinensis* cv. Jinshi > *A. chinensis* cv. Jinlong. Furthermore, compared with the positive inhibitor (acarbose standard, IC_50_ = 4.63 mg/mL), the tested kiwifruits exerted a moderate inhibitory effect towards α-glucosidase. However, kiwifruits exhibited stronger antidiabetic activity than orange, mandarine, apple, banana, pineapple, plum, pear, pomelo, and red grapefruit [8].

## 3. Materials and Methods

### 3.1. Samples and Chemicals

Fourteen different kiwifruits, including eight different cultivars of *Actinidia chinensis*, and three different cultivars of *A. deliciosa*, *A. macrosperma*, *A. polygama*, and *A. arguta*, respectively, were manually collected in different orchards from Sichuan and Shaanxi Province, China. All of the kiwifruit samples were harvested at their commercial maturity (soluble solid content ranging from 6.5% to 7.5%), and then they were ripened at room temperature (20–25 °C) to reach an average firmness of 0.5–1.0 kg/cm^2^. The relevant sample information is shown in Table 1. For each sample, approximately 5 kg of kiwifruits were collected. The whole kiwifruits were washed with distilled water, and divided into two groups. The fresh kiwifruits were used to measure the soluble solids content, dry matter, and ascorbic acid content (first group). The second group was frozen and freeze-dried. Subsequently, the samples were ground to pass through a 60-mesh sieve, and stored at −20 °C for further analysis.

Ascorbic acid, (+)-catechin, (−)-epicatechin, procyanidin B1, procyanidin B2, quercetin, rutin, kaempferol, quercetin-3-*O*-glucoside, quercetin-3-rhamnoside, chlorogenic acid, neochlorogenic acid, cryptochlorogenic acid, caffeic acid, gallic acid, protocatechuic acid, 6-hydroxy-2,5,7,8-tetramethyl chroman-2-carboxylic acid (Trolox), pancreatic lipase, α-glucosidase, *p*-nitrophenyl-α-d-glucopyranoside (PNPG), *p*-nitrophenyl acetate, 2,2-diphenyl-1-picrylhydrazyl (DPPH), 2,2′-azino-bis (3-ethylenzthiazoline-6-sulphonic acid) (ABTS), 2,4,6-tris-2-pyridyl-s-triazine (TPTZ), and the Folin-Ciocalteu reagent were purchased from Sigma-Aldrich (St. Louis, MO, USA). All other reagents and chemicals used were of analytical grade.

### 3.2. Determination of Dry Matter, Soluble Solids Content (SSC), and Ascorbic Acid

The dry matter was determined by drying slices (2 mm) from the middle of the kiwifruit pulp at 105 °C to achieve constantly weight. Furthermore, the pulp of kiwifruit was homogenized in a grinder (Guangming Medical Instrument Co., Ltd, Beijing, China), and the supernatant was collected for analysis of SSC. The SSC was directly measured in the obtained supernatant using a digital refractometer (PAL-1, Atago, Japan). In addition, the content of ascorbic acid was measured according to a previously reported method with minor modifications [36]. Briefly, 3.0 g of fresh samples were homogenized in 50 mL of trichloroacetic acid (TCA, 5%, *w*/*v*) pre-cooled on ice, and centrifuged at 5000× *g* for 10 min at 4 °C. Then, 1 mL of the supernatant, 1 mL of 5% TCA, 1 mL of absolute ethanol, 0.5 mL of 0.4% phosphoric acid-ethanol (*v*/*v*), 1 mL of 1,10-phenanthroline (0.5%, *w*/*v*), and 0.5 mL of ferric trichloride (0.03%, *w*/*v*) were added sequentially, and incubated at 30 °C for 60 min in dark. The absorbance was determined at 534 nm using a Varioskan Flash Multimode Reader (ThermoFisher, Waltham, MA, USA). A standard curve of ascorbic acid was used for calibration.

### 3.3. Extraction of Phenolic Compounds

The extraction of lyophilized kiwifruits was performed according to a previously reported method with minor modifications [37]. Briefly, 1.0 g of lyophilized kiwifruit powder was mixed with 30 mL of 70% acidified methanol (0.1% HCl, *v*/*v*). Then, the mixture was extracted with ultrasound (Kangshijie ultrasound Co., Ltd, Dongguan, Guangzhou, China) (50 kHz, 480 W) for 60 min at room temperature followed by centrifugation (Beckman Coulter, Fullerton, CA, USA) at 6000× *g* for 20 min to collect supernatant, and the extraction was repeated twice under the same extraction conditions. The combined supernatants were evaporated to dryness under a vacuum at 45 °C. The dried residue was then dissolved into 70% of methanol and stored at −20 °C in dark for the further determination of total phenolics, individual phenolic compounds, antioxidant capacities, and inhibitory effects on digestive enzymes. The extract was filtered through a 0.22 μm organic membrane prior to analysis using high performance liquid chromatography (HPLC) (Agilent Technologies, Santa Clara, CA, USA).

### 3.4. Determination of Total Phenolic Content

The total phenolic content (TPC) of kiwifruit extract was measured by the Folin-Ciocalteu assay as described previously with minor modifications [37]. Briefly, 100 μL of each kiwifruit extract or gallic acid standard solution was mixed with 500 μL of Folin-Ciocalteu reagent. Subsequently, 500 μL of sodium carbonate solution (20%, *w*/*v*) was added. After incubation at room temperature for 30 min, the absorbance was measured at 760 nm using a Varioskan Flash Multimode Reader (ThermoFisher, Waltham, MA, USA). The TPC was expressed as milligram gallic acid equivalent per gram of kiwifruit dry weight (mg GAE/g DW).

### 3.5. HPLC Analysis of Individual Phenolic Compounds

Individual phenolic compounds of kiwifruit extract were evaluated by an Agilent 1260 series LC system (Agilent Technologies, Santa Clara, CA, USA) equipped with a diode-array detector (DAD). Chromatographic separations were conducted at 25 °C on a ZORBAX Eclipase XDB-C18 column (250 mm × 4.6 mm, 5 μm). The chromatographic separation was achieved by gradient elution with 0.5% (*v*/*v*) acetic acid solution (A) and acetonitrile (B) according to a previous study, with minor modifications [17]. Briefly, samples were eluted as follows: 0 min, 5% B; 5 min, 5% B; 50 min, 5–20% B; 70 min, 20–70% B; 72 min, 70–5% B; and 72–77 min, 5% B. The flow rate was 0.8 mL/min, and the injection volume was 20 μL for all samples. Detection was made at 280 nm for flavan-3-ols and hydroxybenzoic acids, 320 nm for hydroxycinnamic acids, and 360 nm for flavonols, respectively. Identification of individual phenolic compounds of kiwifruits was carried out by comparing retention times and absorption spectra of commercial standards, and spiking of external reference standards, as well as comparing the information of the same type of samples previously reported [14,17,38]. The identified compounds were quantified by an external calibration method using calibration curves. Fifteen standards, including 4 flavan-3-ols ((+)-catechin, (−)-epicatechin, procyanidin B1, and procyanidin B2), 5 flavonols (quercetin, rutin, kaempferol, quercetin-3-*O*-glucoside, and quercetin-3-rhamnoside), and 6 phenolic acids (chlorogenic acid, neochlorogenic acid, cryptochlorogenic acid, caffeic acid, gallic acid, and protocatechuic acid) were used for the qualitative and quantitative analysis of individual phenolic compounds in the kiwifruit extract. The content of individual phenolic compounds was expressed as microgram per gram dry weight (μg/g DW).

### 3.6. Determination of Antioxidant Capacities of Kiwifruit Extract

#### 3.6.1. ABTS Radical Cation Scavenging Capacity

The ABTS radical cation scavenging capacity of kiwifruit extract was evaluated according to a previously reported method with minor modifications [37]. Briefly, the ABTS radical cation solution was generated by the interaction of 7 mM ABTS solution and 2.45 mM aqueous potassium persulfate at room temperature for at least 16 h in the dark. The ABTS radical cation solution was diluted with phosphate buffer (0.2 M, pH 7.4) to an absorbance of 0.750 ± 0.02 at 734 nm. Then, 200 μL of ABTS radical cation working solution was mixed with 20 μL of each kiwifruit extract at five different concentrations or phosphate buffer as a negative control in a 96-well microplate to react at 30 °C for 20 min. The absorbance at 734 nm was measured. Trolox was used as the standard, and the ABTS radical cation scavenging capacity was expressed as μmol of Trolox equivalent per gram of kiwifruit dry weight (μmol Trolox/g DW), and the IC50 value of ABTS radical cation scavenging was expressed as mg of kiwifruit dry weight /mL (mg/mL).

#### 3.6.2. DPPH Radical Scavenging Capacity

The DPPH radical scavenging capacity was measured according to a previously reported method with slight modifications [37]. Briefly, 25 μL of each kiwifruit extract at five different concentrations or methanol as a negative control was added to 200 μL of DPPH solution (0.35 mM) in a 96-well microplate. The mixed solution was shaken and incubated at room temperature for 30 min. Finally, the absorbance was measured at 517 nm with a blank contain-only DPPH solution and methanol. Trolox was used as the standard, and the DPPH radical scavenging capacity was expressed as μmol of Trolox equivalent per gram of kiwifruit dry weight (μmol Trolox/g DW). The IC_50_ value of DPPH radical scavenging capacity was expressed as mg of kiwifruit dry weight/mL (mg/mL).

#### 3.6.3. Ferric-Reducing Antioxidant Power (FRAP)

The FRAP assay was conducted according to a previously reported method with minor modifications [39]. Briefly, the FRAP working solution contained 300 mM acetate buffer (pH 3.6), 10 mM TPTZ solution in 40 mM HCl, and 20 mM FeCl_3_ solution at a ratio of 10:1:1, and was freshly prepared. The working solution was warmed at 30 °C before usage. Then, 100 μL of each kiwifruit extract was mixed with 3 mL of FRAP solution and incubated at 37 °C for 4 min. Finally, the absorbance was measured at 593 nm. Trolox was used as the standard, and the FRAP was expressed as μmol of Trolox equivalent per gram of kiwifruit dry weight (μmol Trolox/g DW).

### 3.7. Pancreatic Lipase and α-Glucosidase Inhibition of Kiwifruit Extract

The inhibition of pancreatic lipase activity was determined according to a previously reported method with modifications [8]. In brief, the *p*-nitrophenyl acetate (10 mM, dissolved in DMSO) stock solution was diluted with distilled water to reach a final concentration of 2 mM. 100 μL of each kiwifruit extract at five different concentrations, 200 μL of Tris buffer (50 mM, pH 7.4), and 100 μL of lipase solution (5 mg/mL, dissolved in 50 mM pH 7.4 Tris buffer) were mixed and incubated at 37 °C for 10 min. Then, 100 μL of p-nitrophenyl acetate solution (2 mM) was added and incubated at 37 °C for 15 min. Finally, the absorbance was measured at 410 nm. The commercial capsule of orlistat was used as a positive control. The results were expressed as inhibition (%) of pancreatic lipase activity according to the following equation below. The pancreatic lipase inhibitory effect was measured at five different concentrations, and a logarithmic regression curve was established to calculate IC_50_ values (mg of kiwifruit dry weight/mL):

(1) $$\mathrm{Pancreatic}\;\mathrm{lipase}\;\mathrm{inhibition}\%=\left[1-\frac{A_{\mathrm{sample}}-A_{\mathrm{blank}}}{A_{\mathrm{control}}-A_{\mathrm{solvent}\;\mathrm{blank}}}\right]\times 100\%$$

where A_sample_ is the absorbance of the mixture of kiwifruit extract, Tris solution, pancreatic lipase solution, and *p*-nitrophenyl acetate solution; A_blank_ is the absorbance of the mixture of kiwifruit extract, Tris solution, and pancreatic lipase solution; A_control_ is the absorbance of the mixture of methanol, Tris solution, pancreatic lipase solution, and *p*-nitrophenyl acetate solution; and A_solvent blank_ is the absorbance of Tris solution.

The α-glucosidase inhibitory effect was determined according to a previously reported method with some modifications [8]. Briefly, 100 μL of kiwifruit extract at five different concentrations was mixed with 100 μL of α-glucosidase (0.5 U/mL, dissolved in 0.1 M pH 6.8 phosphate buffer), and incubated at 37 °C for 10 min. Then, 25 μL of PNPG solution (4 mM, dissolved in 0.1 M pH 6.8 phosphate buffer) was added to initiate the reaction at 37 °C for 20 min. The absorbance was measured at 405 nm. The α-glucosidase inhibitor (acarbose standard) was used as a positive control. The results were expressed as inhibition (%) of α-glucosidase activity, according to the following equation below. α-Glucosidase inhibitory effect was measured at five different concentrations, and a logarithmic regression curve was established to calculate IC_50_ values (mg of kiwifruit dry weight/mL):

(2) $$\alpha \text{-}\mathrm{Glucosidase}\;\mathrm{inhibition}\%=\left[1-\frac{A_{\mathrm{sample}}-A_{\mathrm{blank}}}{A_{\mathrm{control}}-A_{\mathrm{solvent}\;\mathrm{blank}}}\right]\times 100\%$$

where A_sample_ is the absorbance of the mixture of kiwifruit extract, α-glucosidase solution, and PNPG solution A_blank_ is the absorbance of the mixture of kiwifruit extract, buffer, and α-glucosidase solution; A_control_ is the absorbance of the mixture of buffer, α-glucosidase solution, and PNPG solution; and A_solvent blank_ is the absorbance of buffer solution.

### 3.8. Statistical Analysis

All experiments were conducted in triplicate, and data were expressed in means ± standard deviations. Statistical analysis was performed using SPSS 20.0 software (SPSS 20.0, IBM, Armonk, NY, USA), and the differences among mean values were tested by one-way ANOVA (SPSS 20.0, IBM, Armonk, NY, USA), taking a level of *p* < 0.05 as significant to Duncan’s multiple range test.

## 4. Conclusions

Significant differences were observed in the physicochemical properties and phenolic profiles of different kiwifruits, as well as in the antioxidant capacities and inhibitory effects on digestive enzymes. The total phenolic content of *Actinidia chinensis* cv. Hongshi, *A. chinensis* cv. Jinshi, and *A. chinensis* cv. Jinlong were 16.52 ± 0.26 mg GAE/g DW, 13.38 ± 0.20 mg GAE/g DW, and 11.02 ± 0.05 mg GAE/g DW, respectively, which were much higher than those of the other tested kiwifruits. According to HPLC analysis, procyanidin B1, procyanidin B2, (−)-epicatechin, chlorogenic acid, gallic acid, and quercetin-3-rhamnoside are the main phenolic compounds in kiwifruits. Furthermore, all the tested kiwifruits exerted remarkable antioxidant capacities and inhibitory effects on pancreatic lipase and α-glucosidase. Phenolic compounds, such as chlorogenic acid, procyanidin B1, procyanidin B2, quercetin-3-rhamnoside, and quercetin-3-*O*-glucoside, could be the major contributors toward antioxidant capacities and enzyme inhibition effects of kiwifruits. Indeed, *A. chinensis* cv. Hongshi, *Actinidia chinensis* cv. Jinshi, and *Actinidia chinensis* cv. Jinlong exhibited much better antioxidant capacities and inhibitory effects on digestive enzymes (pancreatic lipase and α-glucosidase) than those of the other tested kiwifruits. Particularly, *A. polygama* showed the highest inhibitory activity on α-glucosidase. Therefore, *Actinidia chinensis* cv. Hongshi, *Actinidia chinensis* cv. Jinshi, and *Actinidia chinensis* cv. Jinlong, as well as *A. polygama* could be important dietary sources of natural antioxidants and natural inhibitors against pancreatic lipase and α-glucosidase, which could be explored further as functional food ingredients for industrial applications.

## Figures and Tables

**Table 1:** Fruit weight, soluble solids content (SSC), dry matter, and ascorbic acid of different kiwifruits.

Samples	Codes	Harvest Date	Regions	Flesh Color	Fruit Weight (g)	SSC (%)	Dry Matter (%)	Ascorbic Acid (mg/100 g FW)
***A. chinensis***								
Hongshi	HS	28 September 2017	Mianzhu, Sichuan, China	Green, and red (middle part)	58.07 ± 2.17 ^g^	16.3 ± 0.1 ^b^	16.49 ± 0.18 ^e^	390.68 ± 4.24 ^a^
Jinshi	JS	15 October 2017	Mianzhu, Sichuan, China	Yellow	107.19 ± 1.48 ^c^	16.1 ± 0.1 ^b,c^	16.55 ± 0.05 ^e^	235.30 ± 8.27^c^
Jinlong	JL	2 November 2017	Xianyang, Shaanxi, China	Yellow	118.25 ± 7.41 ^b^	16.1 ± 0.1 ^c^	15.88 ± 0.10 ^f,g^	335.06 ± 10.39 ^b^
Jinyan	JY	26 October 2017	Chengdu, Sichuan, China	Yellow	88.47 ± 5.30 ^e^	13.1 ± 0.1 ^i^	12.45 ± 0.01 ^j^	97.27 ± 3.59 ^g^
Hongyang	HY	28 September 2017	Cangxi, Sichuan, China	Green, and red (middle part)	95.57 ± 10.22 ^d^	17.6 ± 0.1 ^a^	18.33 ± 0.09 ^b^	107.56 ± 1.34 ^f^
Jinhong	JH	4 September 2017	Dayi, Sichuan, China	Green, and red (middle part)	133.74 ± 5.66 ^a^	16.1 ± 0.1 ^c^	16.63 ± 0.06 ^e^	53.42 ± 1.78 ^j,k^
Honghua	HH	8 September 2017	Pujiang, Sichuan, China	Green, and red (middle part)	107.80 ± 7.74 ^c^	14.3 ± 0.1 ^f^	15.75 ± 0.04 ^f,g^	61.45 ± 1.22 ^i^
Hort16A	HA	26 October 2017	Chengdu, Sichuan, China	Yellow	47.98 ± 4.49 ^h^	15.8 ± 0.3 ^d^	15.67 ± 0.02 ^g^	129.10 ± 3.24 ^e^
***A. deliciosa***								
Cuixing	CX	27 October 2017	Xianyang, Shaanxi, China	Green	78.53 ± 3.16 ^f^	16.2 ± 0.1 ^b,c^	17.54 ± 0.05 ^c^	169.54 ± 2.88 ^d^
Xuxiang	XX	26 October 2017	Chengdu, Sichuan, China	Green	59.90 ± 3.53 ^g^	13.7± 0.1 ^f,h^	14.53 ± 0.09 ^h^	84.38 ± 1.44 ^h^
Hayward	HD	8 August 2017	Xianyang, Shaanxi, China	Green	113.77 ± 8.65 ^b^	13.6 ± 0.1 ^h^	13.48 ± 0.02 ^i^	59.32 ± 1.89 ^i,j^
***A. macrosperma***	AM	24 August 2017	Pujiang, Sichuan, China	Orange	12.79 ± 0.89 ^i^	13.9 ± 0.1 ^g,f^	15.97 ± 0.36 ^f^	61.06 ± 2.91 ^i^
***A. polygama***	AP	24 August 2017	Pujiang, Sichuan, China	Orange	14.31 ± 1.25 ^i^	14.0 ± 0.1 ^g^	17.08 ± 0.15 ^d^	85.94 ± 0.94 ^h^
***A. arguta***	AA	24 August 2017	Pujiang, Sichuan, China	Green	7.10 ± 0.37 ^j^	15.4 ± 0.1 ^e^	20.35 ± 0.31 ^a^	51.32 ± 0.42 ^k^

Values represent mean ± standard deviation. Different letters (a–k) in the same column indicate significant differences at *p* < 0.05 determined by ANOVA, followed by Duncan’s test. The sample codes were the same as in Figure 1.

**Table 2:** Total phenolic content (TPC), ABTS and DPPH radical scavenging capacities, and ferric reducing antioxidant power (FRAP) of different kiwifruits.

Code	TPC (mg GAE/g DW)	ABTS (μmol Trolox/g DW)	DPPH (μmol Trolox/g DW)	FRAP (μmol Trolox/g DW)
HS	16.52 ± 0.26 ^a^	160.36 ± 6.15 ^a^	87.38 ± 4.32 ^a^	149.97 ± 6.98 ^a^
JS	13.38 ± 0.20 ^b^	117.90 ± 2.09 ^b^	71.60 ± 4.04 ^b^	142.58 ± 3.72 ^b^
JL	11.02 ± 0.05 ^c^	90.78 ± 1.29 ^c^	60.14 ± 2.07 ^c^	120.04 ± 1.82 ^c^
JY	6.71 ± 0.12 ^e^	47.92 ± 1.64 ^g^	26.46 ± 0.66 ^e^	54.69 ± 1.48 ^d^
HY	6.22 ± 0.21 ^f^	62.93 ± 1.31 ^e^	22.97 ± 0.53 ^f^	52.73 ± 1.75 ^d^
JH	5.29 ± 0.10 ^h^	38.16 ± 1.03 ^i^	13.12 ± 0.07 ^h^	25.42 ± 0.77 ^f^
HH	4.70 ± 0.10 ^i^	42.68 ± 1.24 ^h^	13.55 ± 0.73 ^h^	29.03 ± 0.47 ^f^
HA	6.60 ± 0.14 ^e^	50.14 ± 0.24 ^g^	26.88 ± 0.09 ^e^	53.84 ± 2.06 ^d^
CX	6.06 ± 0.13 ^f^	55.71 ± 1.32 ^f^	28.10 ± 2.01 ^e^	54.11 ± 0.58 ^d^
XX	5.29 ± 0.10 ^h^	39.52 ± 0.56 ^h,i^	19.54 ± 0.71 ^g^	37.75 ± 1.67 ^e^
HD	3.75 ± 0.09 ^j^	32.95 ± 0.29 ^j^	15.67 ± 0.42 ^h^	26.43 ± 0.97 ^f^
AM	8.15 ± 0.19 ^d^	84.07 ± 0.61 ^d^	39.69 ± 1.85 ^d^	54.75 ± 0.37 ^d^
AP	5.57 ± 0.12 ^g^	64.71 ± 1.40 ^e^	21.47 ± 0.17 ^f,g^	38.18 ± 0.77 ^e^
AA	4.71 ± 0.18 ^i^	55.81 ± 0.71 ^f^	14.08 ± 0.20 ^h^	27.24 ± 0.41 ^f^

Values represent mean ± standard deviation. Different letters (a–j) in the same column indicate significant differences at *p* < 0.05 determined by ANOVA, followed by Duncan’s test. The sample codes were the same as in Table 1.

**Table 3:** Calibration data and limits of detection (LOD) for the 15 investigated phenolic compounds in kiwifruit.

Compounds	Regression Equation	Linear Range (μg/mL)	R^2^	LOD (μg/mL)
gallic acid	Y = 54.107X − 8.928	0.50–10.00	0.9998	0.16
protocatechuic acid	Y = 28.455X + 0.213	0.78–10.00	1.0000	0.26
neochlorogenic acid	Y = 74.454X − 4.940	0.22–40.00	1.0000	0.07
procyanidin B1	Y = 13.806X − 11.478	2.78–250.00	1.0000	0.92
chlorogenic acid	Y = 71.270X − 3.391	0.10–40.00	1.0000	0.30
(+)-catechin	Y = 16.756X − 9.765	2.00–40.00	0.9999	0.70
cryptochlorogenic acid	Y = 59.701X + 0.326	0.19–1.50	1.0000	0.06
caffeic acid	Y = 102.38X − 5.749	0.30–12.00	0.9999	0.10
procyanidin B2	Y = 14.637X − 2.1539	0.71–50.00	0.9999	0.23
(−)-epicatechin	Y = 20.575X + 0.264	0.50–25.00	1.0000	0.16
rutin	Y = 30.337X − 6.2411	1.00–10.00	0.9998	0.03
quercetin-3-*O*-glucoside	Y = 39.956X − 3.1422	0.44–20.00	0.9998	0.12
quercetin-3-rhamnoside	Y = 34.673X − 8.953	0.75–15.00	0.9993	0.25
quercetin	Y = 67.113X − 22.838	0.80–8.00	0.9992	0.26
kaempferol	Y = 94.982X − 16.021	0.33–10.00	0.9998	0.11

**X**, concentration (μg/mL); **Y**, peak area.

**Table 4:** Content of phenolic compounds in different kiwifruits.

Code	Flavan-3-ols (μg/g DW)				Phenolic Acids (μg/g DW)					Flavonols (μg/g DW)		
	PB1	PB2	EC	Ca	GA	PA	CHL	NCHL	CA	QRha	QGlu	Ru
HS	446.81 ± 1.51 ^a^	182.11 ± 1.16 ^a^	162.61 ± 0.99 ^a^	32.08 ± 0.35 ^c^	24.98 ± 0.19 ^d^	15.76 ± 0.18 ^a^	235.75 ± 5.44 ^a^	39.26 ± 1.41 ^c^	n.d	41.94 ± 1.44 ^a^	6.16 ± 0.05 ^c^	n.d
JS	201.69 ± 2.28 ^b^	n.d	60.70 ± 1.07 ^b^	n.d	53.76 ± 0.43 ^a^	6.55 ± 0.12 ^b^	78.32 ± 1.12 ^f^	107.06 ± 1.74 ^b^	n.d	4.59 ± 0.09 ^g^	n.d	n.d
JL	145.68 ± 3.12 ^f^	20.91 ± 0.53 ^h^	38.98 ± 0.94 ^e^	n.d	26.69 ± 0.45 ^c^	n.d	55.22 ± 0.48 ^h^	133.72 ± 3.98 ^a^	n.d	n.d	n.d	n.d
JY	195.99 ± 4.29 ^c^	125.38 ± 2.00 ^c^	43.41 ± 0.98 ^c^	n.d	49.13 ± 0.27 ^b^	n.d	121.41 ± 0.92 ^c^	6.29 ± 0.11 ^f^	n.d	4.73 ± 0.11 ^g^	n.d	n.d
HY	132.33 ± 1.33 ^g^	78.62 ± 1.18 ^e^	45.60 ± 0.97 ^c^	n.d	14.62 ± 0.16 ^g^	n.d	97.22 ± 1.60 ^e^	15.86 ± 1.04 ^e^	n.d	21.57 ± 1.08 ^c^	n.d	n.d
JH	188.54 ± 1.73 ^d^	n.d	22.97 ± 0.15 ^h^	n.d	21.25 ± 0.35 ^f^	n.d	184.15 ± 1.97 ^b^	8.27 ± 0.14 ^f^	n.d	9.16 ± 0.21 ^f^	n.d	n.d
HH	155.11 ± 2.32 ^e^	n.d	n.d	n.d	12.17 ± 0.25 ^h^	n.d	37.78 ± 1.19 ^i^	6.53 ± 0.17 ^f^	n.d	7.88 ± 0.04 ^f^	n.d	n.d
HA	203.68 ± 4.27 ^b^	114.95 ± 1.84 ^d^	60.56 ± 1.77 ^b^	n.d	22.68 ± 0.30 ^e^	n.d	103.65 ± 1.47 ^d^	17.78 ± 1.53 ^d,e^	n.d	n.d	n.d	n.d
CX	64.60 ± 1.33 ^k^	17.88 ± 0.66 ^h^	13.60 ± 0.30 ^i^	n.d	5.64 ± 0.13 ^k^	n.d	20.94 ± 0.61 ^k^	n.d	n.d	16.19 ± 0.53 ^e^	n.d	n.d
XX	118.58 ± 1.72 ^h^	146.28 ± 2.65 ^b^	27.96 ± 0.63 ^g^	14.71 ± 0.59 ^d^	9.12 ± 0.08 ^j^	n.d	75.25 ± 2.52 ^f^	n.d	n.d	32.25 ± 0.60 ^b^	5.10 ± 0.12 ^c^	n.d
HD	91.03 ± 1.44 ^j^	45.54 ± 0.87 ^g^	n.d	n.d	9.74 ± 0.13 ^i^	n.d	60.77 ± 0.49 ^g^	n.d	n.d	8.55 ± 0.17 ^f^	n.d	n.d
AM	107.98 ± 1.53 ^i^	112.74 ± 2.19 ^d^	31.48 ± 1.60 ^f^	28.72 ± 1.43 ^c^	n.d	n.d	27.12 ± 1.07 ^j^	n.d	40.97 ± 1.15 ^a^	8.69 ± 0.15 ^f^	n.d	17.58 ± 0.58 ^b^
AP	n.d	81.42 ± 0.98 ^e^	26.12 ± 0.63 ^g^	43.30 ± 2.47 ^b^	9.90 ± 0.09 ^i^	12.19 ± 0.24 ^c^	63.50 ± 0.89 ^g^	n.d	2.69 ± 0.03 ^c^	16.43 ± 0.41^d,e^	108.60 ± 0.92 ^a^	48.23 ± 0.38 ^a^
AA	58.72 ± 0.74 ^l^	65.17 ± 0.45 ^f^	n.d	132.08 ± 1.16 ^a^	n.d	n.d	7.70 ± 0.04 ^l^	21.14 ± 1.11 ^d^	22.56 ± 0.90 ^b^	17.63 ± 0.59 ^d^	11.02 ± 0.20 ^b^	8.27 ± 0.08 ^c^

**PB1**, procyanidin B1; **PB2**, procyanidin B2; **EC**, (−)-epicatechin; **Ca**, (+)-catechin; **GA**, gallic acid; **PA**, protocatechuic acid; **CHL**, chlorogenic acid; **NCHL**, neochlorogenic acid; **CA**, caffeic acid; **QRha**, Quercetin-3-rhamnoside; QGlu, quercetin-3-*O*-glucoside; **Ru**, rutin; **n.d**, not detected; each value represents the mean ± standard deviation. Different letters in the same column indicate significant differences at *p* < 0.05 determined by ANOVA, followed by Duncan’s test. The sample codes are the same as in Table 1.
